# Exploration of the core pathway of inflammatory bowel disease complicated with metabolic fatty liver and two-sample Mendelian randomization study of the causal relationships behind the disease

**DOI:** 10.3389/fimmu.2024.1375654

**Published:** 2024-04-18

**Authors:** Zhiyuan Wei, Jiangbin Wang

**Affiliations:** Department of Digestive, China-Japan Union Hospital of Jilin University, Changchun, Jilin, China

**Keywords:** inflammatory bowel disease, nonalcoholic fatty liver disease, IL-17, chemokine, therapy

## Abstract

**Background:**

Inflammatory bowel disease (IBD) is often associated with complex extraintestinal manifestations. The incidence of nonalcoholic fatty liver disease (NAFLD) in IBD populations is increasing yearly. However, the mechanism of interaction between NAFLD and IBD is not clear. Consequently, this study aimed to explore the common genetic characteristics of IBD and NAFLD and identify potential therapeutic targets.

**Materials and methods:**

Gene chip datasets for IBD and NAFLD were obtained from the Gene Expression Omnibus (GEO) database. Weighted gene co-expression network analysis (WGCNA) was performed to identify modules in those datasets related to IBD and NAFLD. ClueGO was used for biological analysis of the shared genes between IBD and NAFLD. Based on the Human MicroRNA Disease Database (HMDD), microRNAs (miRNAs) common to NAFLD and IBD were obtained. Potential target genes for the miRNAs were predicted using the miRTarbase, miRDB, and TargetScan databases. Two-sample Mendelian randomization (MR) and two-way MR were used to explore the causal relationship between Interleukin-17 (IL-17) and the risk of IBD and NAFLD using data from GWAS retrieved from an open database.

**Results:**

Through WGCNA, gene modules of interest were identified. GO enrichment analysis using ClueGO suggested that the abnormal secretion of chemokines may be a common pathophysiological feature of IBD and NAFLD, and that the IL-17-related pathway may be a common key pathway for the pathological changes that occur in IBD and NAFLD. The core differentially expressed genes (DEGs) in IBD and NAFLD were identified and included COL1A1, LUM, CCL22, CCL2, THBS2, COL1A2, MMP9, and CXCL8. Another cohort was used for validation. Finally, analysis of the miRNAs identified potential therapeutic targets. The MR results suggested that although there was no causal relationship between IBD and NAFLD, there were causal relationships between IL-17 and IBD and NAFLD.

**Conclusion:**

We established a comorbid model to explain the potential mechanism of IBD with NAFLD and identified the chemokine-related pathway mediated by cytokine IL-17 as the core pathway in IBD with NAFLD, in which miRNA also plays a role and thus provides potential therapeutic targets.

## Introduction

Inflammatory bowel disease (IBD) refers to a group of immune-related chronic systemic inflammatory diseases, often with complex extraintestinal manifestations that involve the liver (1), with nonalcoholic fatty liver disease (NAFLD) and autoimmune liver disease the most common (2, 3). Among these latter two, NAFLD is the most common chronic liver disease globally, and its incidence in IBD populations is increasing yearly (4), with many IBD patients characterized by weight loss or even severe emaciation due to diarrhea, the malabsorption of nutrients, and systemic inflammatory responses. Traditionally, a fatty liver in IBD patients was generally not a concern. However, more and more epidemiological investigations are showing that, compared with the general population, the incidence of NAFLD in IBD patients is not decreased, but is in fact increased (5–7), and that IBD-induced NAFLD not only increases the health burden of patients, but also leads to a poor prognosis and aggravation of the IBD conditions. Therefore, it would be of interest to study the changes in gene expression in IBD and NAFLD to study their relation to this condition.

Currently, research into the mechanism of IBD combined with NAFLD is mainly carried out from different perspectives or viewpoints. One perspective is that metabolic disorders are one of the main causes of IBD with NAFLD, because metabolic disorders can affect the intestinal barrier function and cause intestinal inflammation, which can promote the occurrence and development of IBD combined with NAFLD (8), and hence these are a key focus of research. Another focus of research is based around gut microbiota alterations because these are also thought to play a role in the disease process of IBD combined with NAFLD (9). In particular, as IBD and NAFLD display similar changes in gut microbiota composition (10), it is considered that the change in intestinal flora may be one of the main causes of IBD combined with NAFLD. Another study (11) suggested that changes in the composition of the intestinal flora in IBD patients lead to changes in the microbial metabolites, which can further aggravate damage to the intestinal barrier and thus may affect the liver through leakage through the damaged intestinal barrier, leading to NAFLD. A third research perspective is that the effects of drugs used for treating IBD, such as hormones and biological agents, on the liver are of concern and should be considered. Although this viewpoint is controversial, there are numerous studies demonstrating the association between the drugs used for treating IBD and NAFLD (12, 13). A fourth viewpoint is that because both diseases share most of the same characteristics of being immune-mediated inflammatory diseases, the inflammatory response may also play a role in IBD with NAFLD (14). This viewpoint may be backed up by the elevated levels of pro-inflammatory cytokines, such as IL-6 and TNF-α, and decreased levels of anti-inflammatory cytokines, such as IL-10, found in the peripheral blood of patients (15–17). Further, it has been reported that long-term exposure of the liver to chronic and recurrent inflammation makes it prone to the development of NAFLD (18). Further, animal experiments showed that mice treated with dextran sulfate sodium (DSS) and a high fat diet (HFD) developed more severe liver steatosis and liver inflammation than those with a HFD alone (19, 20).

In summary, a number of recent studies have explored the relationship between IBD and NAFLD from different viewpoints and in terms of metabolic disorders, intestinal flora changes, drugs for IBD treatment, and immune inflammatory responses; yet the specific molecular mechanism of IBD combined with NAFLD and whether gene polymorphisms play a role are still unclear. In recent years, the emergence of the concept of the gut–liver axis has prompted researchers and others to increasingly consider the gut and liver as a whole, and to note that some liver diseases share the same pathogenesis with intestinal diseases (21, 22). A possible example is IBD and NAFLD. Although these appear to be two contradictory diseases, there is an increased incidence of NAFLD in IBD patients. In order to explain the trigger factors behind this, the present study sought to explore the common biological mechanisms in NAFLD and IBD by comparing liver biopsies and intestinal biopsies of NAFLD and IBD patients. To assess the relationships among the potential causes, we decided to perform a Mendelian randomization (MR) study. MR has become popular in recent years as a reliable method for inferring potential causal relationships, and has been applied using single nucleotide polymorphisms (SNPs) as instrumental variables (IVs) to assess the causal relationships between exposure factors and outcomes. Of interest in the present study, it was believed that MR using genetic variants strongly associated with different exposure factors as instrumental variables could infer the causal relationships between the exposure factors and study outcomes. Therefore, this study used bioinformatics technology to explore the molecular mechanism of IBD combined with NAFLD by analyzing the shared gene characteristics of IBD and NAFLD, and then used two-sample MR and two-way MR to explore the causal relationship between the identified gene polymorphisms and IBD and NAFLD.

## Materials and methods

### Downloading and preprocessing the study datasets

NAFLD and IBD gene expression profiles were first retrieved from The Cancer Genome Atlas (TCGA) by considering the terms “nonalcoholic fatty liver disease “ and “inflammatory bowel disease”, and then analyzed in the Gene Expression Omnibus (GEO) database to identify datasets with these profiles. The GSE126848 dataset in GEO was chosen as the NAFLD dataset, and included 57 samples, comprising 14 controls, 12 healthy obesity, 15 NAFLD, and 16 nonalcoholic steatohepatitis (NASH) cases. The GSE193677 RNA-seq dataset in GEO was used as the IBD dataset, containing a total of 1173 samples. Also, the GSE179285 dataset was used as the IBD group validation set, and GSE89632 and GSE130970 were used as the NAFLD group validation set. The original data were used for the subsequent analysis after background correction, normalization, and relative expression calculation.

### Screening the characteristic genes through weighted gene co-expression network analysis (WGCNA)

The NAFLD and IBD datasets GSE126848 and GSE179285 were analyzed by weighted gene correlation network analysis (WGCNA). It was found that the clustering effect of the samples was good, and the threshold for the cut-off line was set at 30, while a soft threshold of 1:20 was used for the topological calculations, and the optimal soft threshold was determined to be 6. Next, gene clustering analysis was performed, and the gene significance (GS) and module membership (MM) of each module were calculated and plotted in a scatter plot format. Finally, Pearson correlation analysis was conducted to estimate the correlation between disease occurrence and the merged modules.

### Selection of the characteristic genes and functional identification

Modules that were highly correlated with NAFLD and IBD were selected, and a Venn diagram was utilized to identify the shared genes that overlapped between the modules that were positively associated with NAFLD and IBD. The non-redundant GO and KEGG terms were also classified and visually arranged as functionally grouped networks using the Cytoscape plugin unit ClueGO. Subsequently, we used ClueGO for biological analysis of the shared genes to identify their potential roles in NAFLD and IBD.

### Identification, functional analysis, and external verification of the differentially expressed genes

The differentially expressed genes (DEGs) were identified based on significance analysis using the limma package and considering adjusted P < 0.05 and |log2FC|>0 values, and then the “pheatmap” and “ggplot2” R packages were used to generate volcano maps and DEG expression heat maps. The genes that were elevated and decreased in both NAFLD and IBD were identified through plotting a Venn diagram. GO and KEGG enrichment analyses of the DEGs were performed using the Cytoscape plugin unit ClueGO, and the unique gene signatures of NAFLD and IBD were distinguished by developing a protein–protein interaction (PPI) network and by clustering analysis. The PPI network was built by interrogating the STRING database of known and predicted protein-protein interactions and selecting those with a minimum required interaction score of 0.4. Cluster analysis was performed using the “MCODE” algorithm with the default parameters provided in the Cytoscape software (version 3.9.1). The datasets GSE179285, GSE89632, and GSE130970 were used as external verification sets, and an R4.2.1 block diagram was used to verify the expressions of the characteristic genes in the external verification set.

### Mendelian randomization

All the data in this study were available and used in an open database. Two-sample and bidirectional MR were applied to explore the causal relationship between the identified target of interest from the above analysis, which was interleukin-17 (IL-17), and the risk of IBD and NAFLD, and the SNPs were defined as the IVs. A genome-wide significance level of p < 5×10^-8^, minor allele frequency < 0.01, and clumping algorithm with a cutoff of r2 = 0.001 and kb = 10000 were used to avoid linkage disequilibrium (LD). The strength of the genetic variance of each exposure was estimated using R2 and F-statistics. IVs with F-statistics over 10 were used in the subsequent analyses to avoid weak instrument bias. The genetic data were obtained from publicly available genome-wide association studies (GWAS) data sources. The IL-17, IBD, and NAFLD datasets selected for analysis, and the exposure and outcome data are detailed in **Table 1**. Inverse-variance weighted (IVW) meta-analysis with a multiplicative random model was used as the major analysis methodology for causal estimation. Cochran’s Q test was performed to detect heterogeneity among the genetic variants. The weighted median model, MR−Egger regression model, and Mendelian randomization pleiotropy residual sum and outlier were performed for the sensitivity analyses and to detect the existence of horizontal pleiotropy that would violate the main MR assumptions. The MR analysis was based on the “TwoSampleMR” software package and used inverse variance weighting (IVW) to assess the association between IL-17 and the risk of IBD and NAFLD. Also, MR-Egger was used for additional sensitivity analyses.

**Table 1:** MR Exposure and outcome data.

Phenotype	Sample size	Year	Ethnicity	Number of SNPs	Web source
IBD	12,924	2020	European	6,490,614	https://gwas.mrcieu.ac.uk/datasets/met-d-ApoB/
IBD	484,598	2021	European	9,886,868	https://www.ebi.ac.uk/gwas/studies/GCST90038683
NAFLD	778,614	2021	European	6,787,908	https://www.ebi.ac.uk/gwas/studies/GCST90091033
IL17A	5,362	2022	European	7,506,463	https://www.ebi.ac.uk/gwas/studies/GCST90087632
IL17B	5,358	2022	European	7,506,463	https://www.ebi.ac.uk/gwas/efotraits/EFO_0008174
IL17F	5,362	2022	European	7,506,463	https://www.ebi.ac.uk/gwas/studies/GCST90088065

### Statistical analyses

R software 4.2.1 was used for the statistical analysis. Wilcoxon’s test was used to analyze the significance of the differences between the two groups. Spearman’s correlation test was used to determine the correlations between variables. Statistically significant results were defined as those with a *p*-value < 0.05.

## Results

### Analysis of the co-expressed genes in IBD and NAFLD

**Figure 1:**
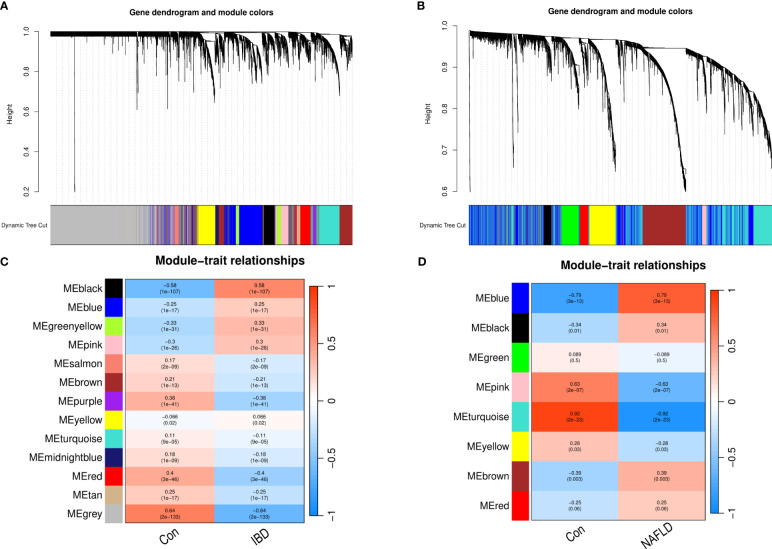
Weighted gene co-expression network analysis (WGCNA). **(A)** Cluster tree of the IBD co-expressed genes. **(B)** Cluster tree of the NAFLD co-expressed genes. **(C)** Module–feature relationships in IBD. Each cell contains the corresponding correlation and p-value. **(D)** Module–feature relationships in NAFLD. Each cell contains the corresponding correlation and p-value. IBD, Inflammatory bowel disease; NAFLD, Nonalcoholic fatty liver disease.

WGCNA was performed on CD and UC in the ileum, colon (including right hemicolon, transverse colon, and left hemicolon), and rectum (**Supplementary Figure 1**). KEGG and GO enrichment analysis were performed on the identified modules (**Supplementary Figure 2**). UC and CD were found to cluster similar modules in different tissues, with little difference observed in the enrichment analysis results, supporting their incorporation into multi-tissue networks. Through WGCNA, 13 gene modules were identified in the GSE193677 dataset and 8 gene modules in GSE126848 related to IBD and NAFLD. The Spearman correlation coefficients were used to draw a correlation heat map between the diseases and each module. Here, the “black” module in the analysis had the highest positive correlation with IBD (r = 0.58), and included 419 genes (see **Figures 1A, B**); while the “blue” module had the highest positive correlation with NAFLD (r = 0.79), and included 1556 genes (see **Figures 1C, D**).

### Common characteristic genes and their functions in IBD and NAFLD

**Figure 2:**
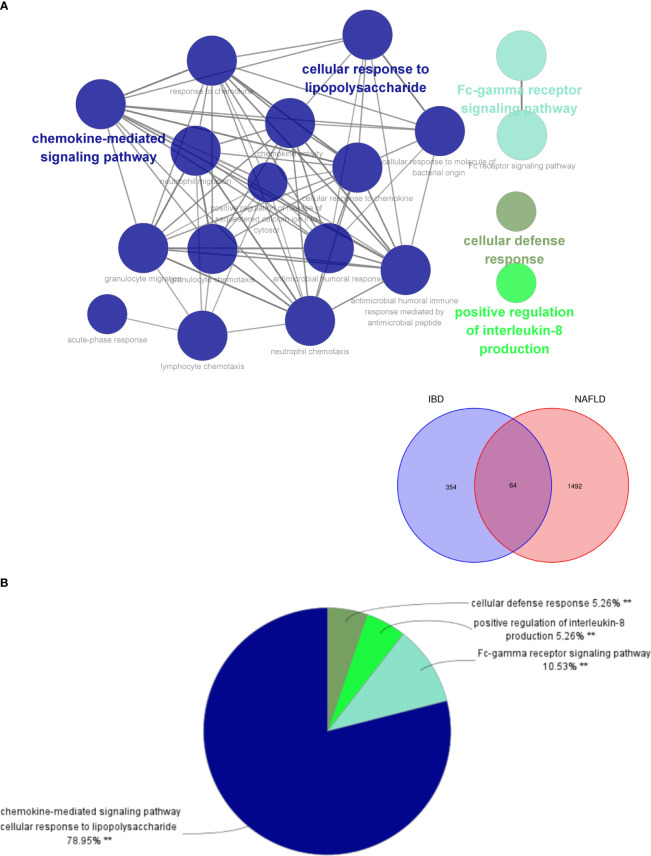
ClueGO enrichment analysis. **(A)** Interactive network of GO terms groups generated by the Cytoscape plugin ClueGO, and **(B)** proportion of each GO terms group in the total. GO, gene ontology. **p<0.05.

By analyzing the two modules with the most positive and highest correlations between the two diseases, a total of 64 genes were identified through them intersecting and overlapping in the relevant core modules of IBD and NAFLD and these were collectively denoted as Gene Set 1 (GS1). ClueGO was used to explore the potential functions of the genes in GS1 through GO enrichment and KEGG enrichment analyses. The top three significantly enriched GO terms in terms of the biological process (BP) were the “chemokine-mediated signaling pathways”, “cellular responses to lipopolysaccharide”, and “fc-gamma receptor signaling pathways” (**Figure 2A**). The chemokine-mediated signaling pathways and cellular responses to lipopolysaccharide accounted for 78.95% of all the GO terms (**Figure 2B**).

**Figure 3:**
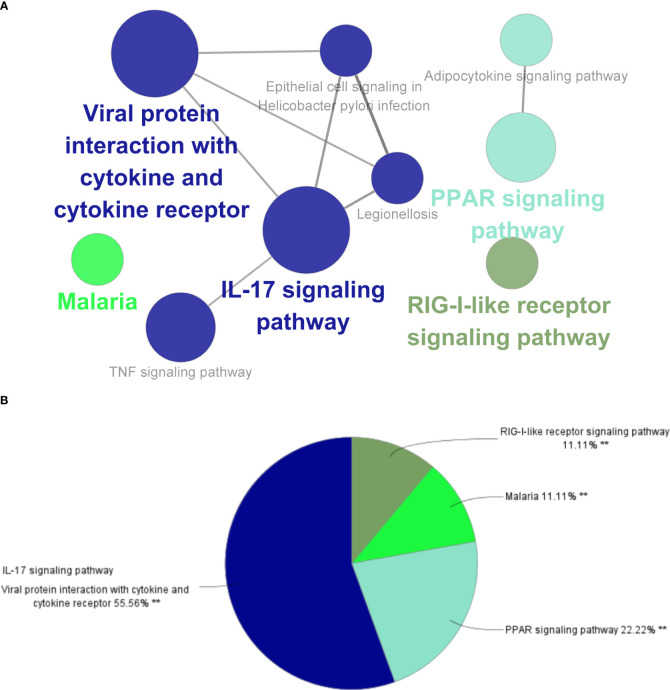
ClueGO enrichment analysis. **(A)** Interactive network of KEGG terms groups generated by the Cytoscape plugin ClueGO, and **(B)** proportion of each KEGG terms group in the total. KEGG, Kyoto Encyclopedia of Genes and Genomes. **p<0.05.

The top three significantly enriched KEGG terms were the “interaction of viral proteins with cytokines and cytokine receptors”, “IL-17 signal pathway”, and “PPAR signal pathway” (**Figure 3A**), with the signaling pathways accounting for 55.56% of all the KEGG terms (**Figure 3B**).

At the same time, in order to elucidate the biological functions and pathways involved in IBD and NAFLD, KEGG and GO enrichment analyses were performed on all the identified modules. It was found that the enrichment analysis results of the brown module in IBD had the highest similarity to G1, while the enrichment results of the turquoise module in NAFLD had the highest similarity to G1 (**Supplementary Figures 3**-**6**).

### Different characteristic genes and their functions in IBD and NAFLD

**Figure 4:**
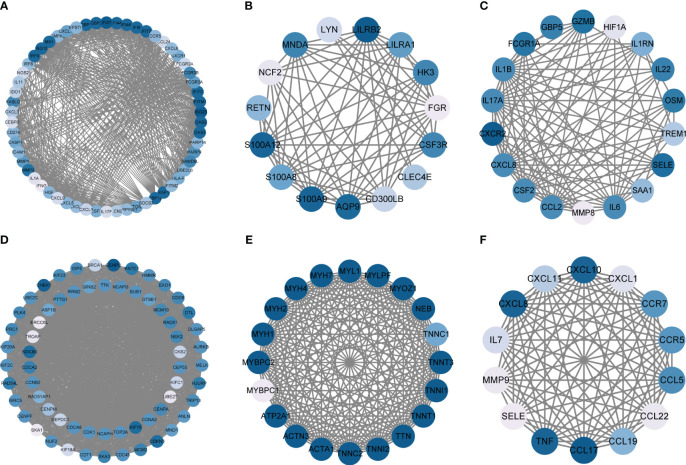
PPI network. **(A–C)** Clusters 1–3 obtained from the black module of IBD, **(D–F)** Clusters 1–3 obtained from the blue modules in patients with NAFLD. PPI, Protein–protein network; IBD, inflammatory bowel disease; NAFLD, nonalcoholic fatty liver disease.

**Figure 5:**
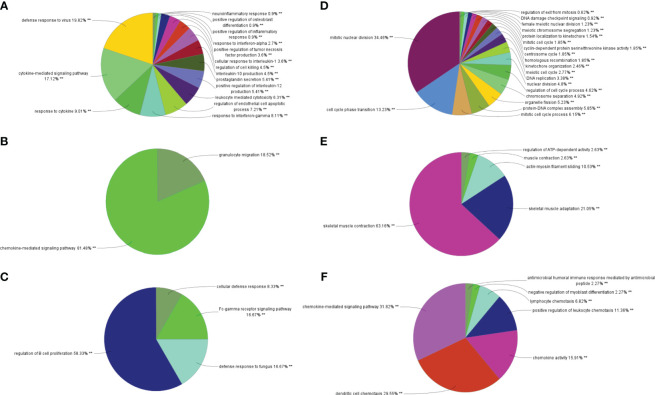
Biological process analysis of GO enrichment. **(A–C)** GO biological process analysis of three gene clusters in IBD, **(D–F)** GO biological process analysis of three NAFLD gene clusters. GO, gene ontology; IBD, inflammatory bowel disease; NAFLD, nonalcoholic fatty liver disease. **p<0.05.

**Figure 6:**
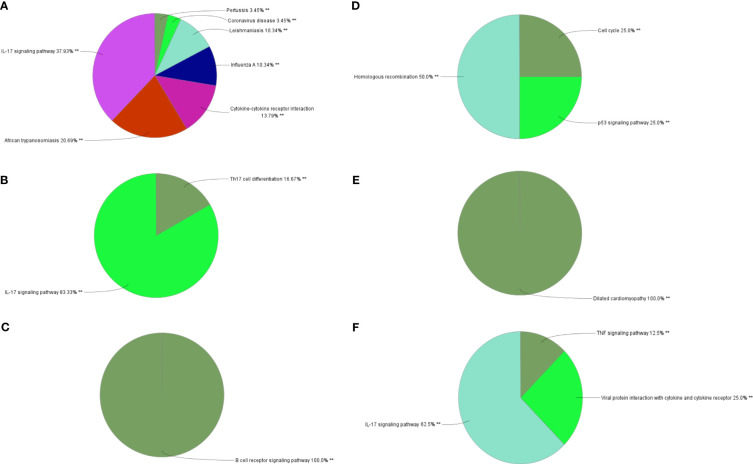
Biological process analysis of KEGG enrichment. **(A–C)** KEGG enrichment analysis of three gene clusters in IBD. KEGG enrichment analysis of three gene clusters of **(D–F)** in NAFLD. KEGG: Kyoto Encyclopedia of Genes and Genomes; IBD, inflammatory bowel disease; NAFLD, nonalcoholic fatty liver disease. **p<0.05.

Through MCODE clustering analysis of the PPI in IBD, three clusters were identified. It was found that Cluster 1 had 58 nodes and 595 edges (score: 20877) (**Figure 4A**), Cluster 2 had 18 nodes and 95 edges (score: 11176) (**Figure 4B**), and Cluster 3 had 15 nodes and 69 edges (score: 9857) (**Figure 4C**). According to the results of the GO enrichment analysis, Cluster 2 was found to be mainly related to the chemokine-mediated signaling pathway, while the chemokine-mediated signaling pathway in Cluster 1 was second only to the defense response to viruses. Therefore, it was speculated that Cluster 2 belonged to the common genes segment of IBD and NAFLD (**Figure 5B**), while Cluster 1 belonged to the similar gene fragments of IBD and NAFLD, and Cluster 3 was considered to relate to the unique genetic features of IBD (**Figures 5A, C**). The MCODE clustering analysis of NAFLD found that Cluster 1 had 63 nodes and 1867 edges (score: 60226) (**Figure 4D**), Cluster 2 had 20 nodes and 185 edges (score: 19474) (**Figure 4E**), and Cluster 3 had 14 nodes and 87 edges (score: 11600) (**Figure 4F**). According to the results of the GO enrichment analysis, Cluster 3 was found to be mainly related to the chemokine-mediated signaling pathway. Therefore, it was speculated that Cluster 3 belonged to the common gene fragments of IBD and NAFLD (**Figure 5F**), while the other two clusters were considered to be unique genetic features of NAFLD (**Figures 5D, E**). The KEGG enrichment analysis validated our conclusions and indicated that the IL-17 signaling pathway plays an important role in both IBD and NAFLD processes (**Figure 6**).

In order to further confirm the above results, WGCNA analysis was performed on patients with IBD and NAFLD only (**Supplementary Figure 7**), and KEGG and GO enrichment analysis were performed on the most relevant modules (**Supplementary Figure 8**). The results were found to partially overlap those of the G1 analysis.

### DEGs and their functional analysis in IBD and NAFLD

**Figure 7:**
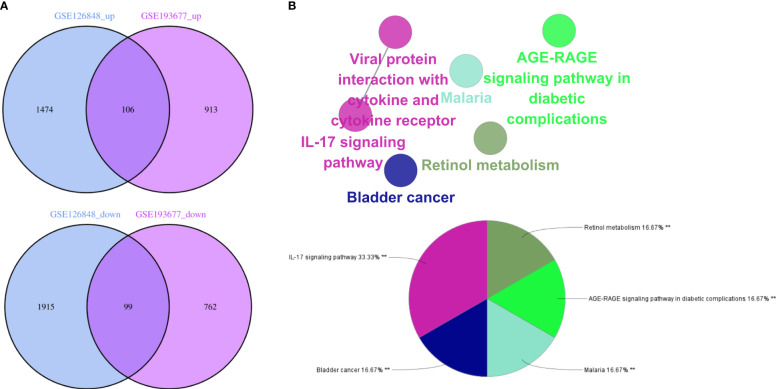
Identification of the common DEGs and ClueGO enrichment analysis. **(A)** Venn diagrams of the upregulated and downregulated genes in IBD and NAFLD, **(B)** Interaction network of KEGG terms generated by the Cytoscape plugin ClueGO and the proportion of KEGG terms in the total. KEGG: Kyoto Encyclopedia of Genes and Genomes; IBD, inflammatory bowel disease; NAFLD, nonalcoholic fatty liver disease. **p<0.05.

**Figure 8:**
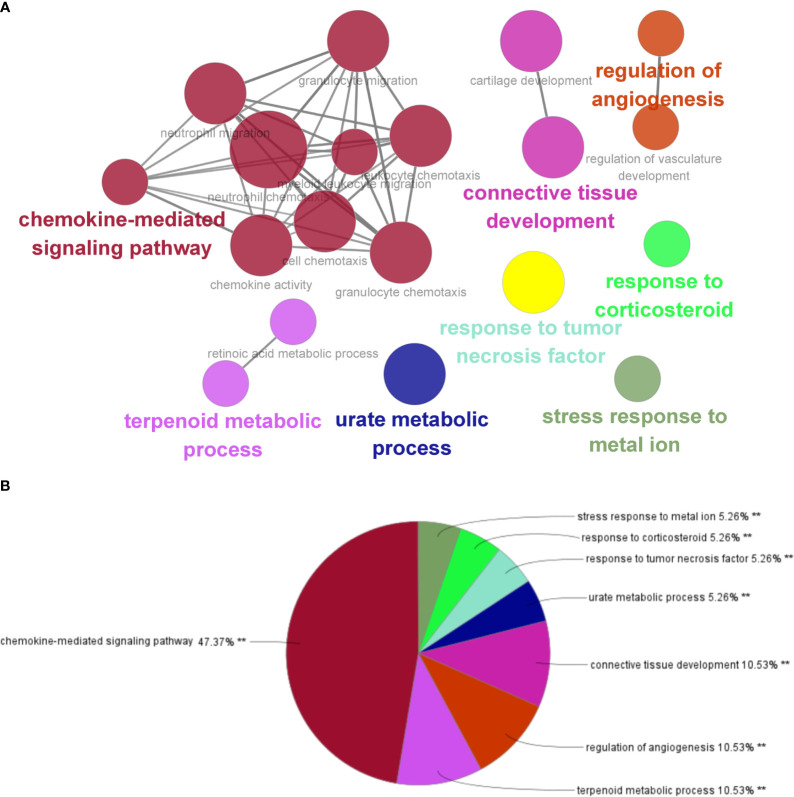
Interaction network of KEGG terms generated by the Cytoscape plugin ClueGO and the proportion of KEGG terms in the total. GO, Gene ontology; IBD, inflammatory bowel disease; NAFLD, nonalcoholic fatty liver disease. **p<0.05.

Overall, 106 upregulated genes and 99 downregulated genes were identified among the DEGs between the GSE193677 and GSE126848 datasets and were collectively denoted as Gene Set 2 (GS2) (**Figure 7A**). KEGG enrichment analysis showed that the “interaction between viral proteins and cytokines and cytokine receptors” and “IL-17 signal pathway” were highly consistent with the results from WGCNA (**Figure 7B**). Meanwhile, the results of the GO enrichment analysis were similar to those from the GO enrichment analysis of GS1 (**Figure 8**).

**Figure 9:**
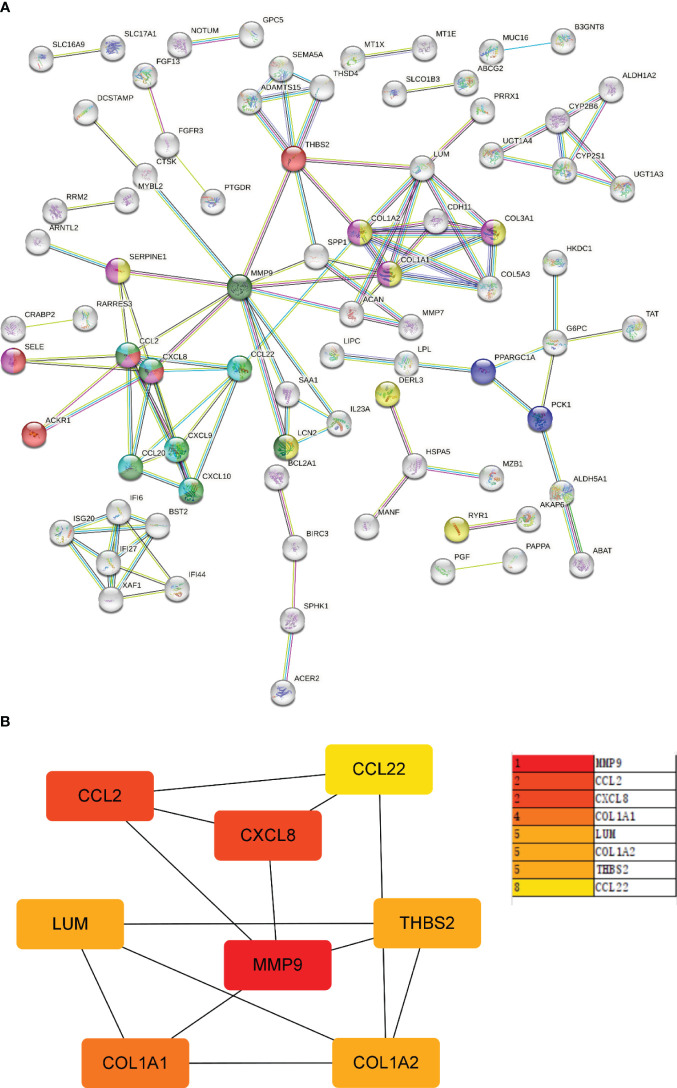
PPI network and core gene network. **(A)** PPI network; **(B)** Core gene network and ranking.

Next, the PPI network for GS2 was established at the protein level and visualized by Cytoscape software (**Figure 9A**). The core genes were identified by cytoHubba, a plug-in based on Cytoscape, which is typically used for network topology analysis and node centrality analysis. The plug-in assigns a value to each gene through the topology network algorithm, and then finds its key genes (hub genes) and subnetworks. By applying the maximum clique centrality (MCC) algorithm, we identified eight core genes, namely COL1A1, LUM, CCL22, CCL2, THBS2, COL1A2, MMP9, and CXCL8, and these were collectively denoted as Gene Set 3 (GS3) (**Figure 9B**).

**Figure 10:**
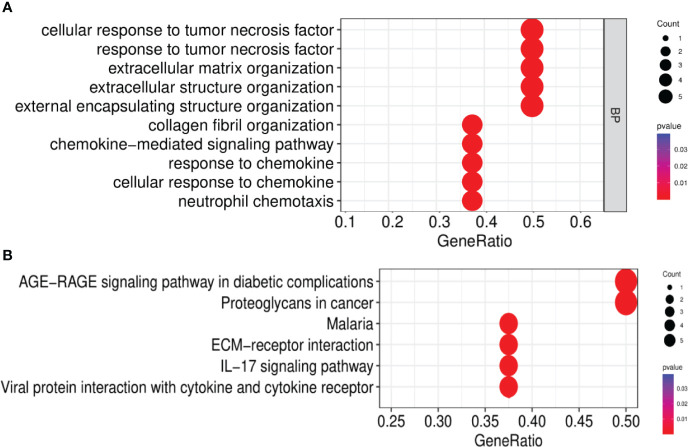
GO and KEGG enrichment analysis. **(A)** GO enrichment analysis (BP), **(B)** KEGG enrichment analysis.

After the GO enrichment analysis and KEGG enrichment analysis of GS3, it was found that the “chemokine-mediated signaling pathway”, “interaction of viral protein with cytokines and cytokine receptors”, and “IL-17 signaling pathway” were all significantly enriched in GS3 (**Figures 10A, B**). This suggested that these three pathways may play an important role in the disease progression of IBD and NAFLD.

**Figure 11:**
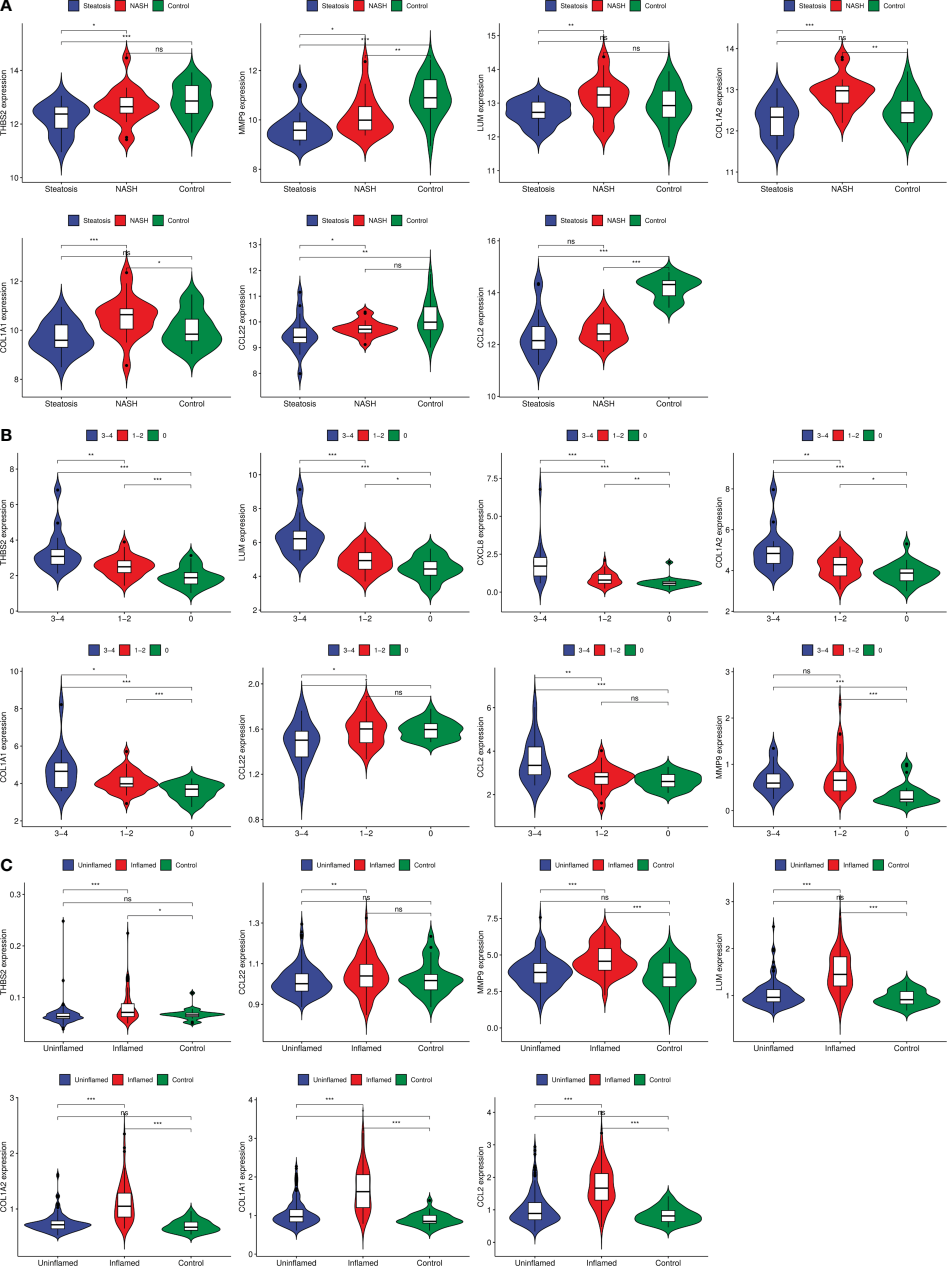
Gene expression of each group at different stages of IBD progression. **(A)** GSE89632 gene expression, **(B)** GSE130970 gene expression, **(C)** GSE179285 gene expression. ***:p< 0.001, **:p<0.01, *:p<0.05, ns:p>0.05.

The GSE179285, GSE89632, and GSE130970 datasets were used as validation groups to verify the expression of GS3 in the progression of IBD and NAFLD (**Figure 11**). The results showed that the gene expression in GS3 was significantly increased in IBD patients during intestinal inflammation, and in the process of NAFLD complicated with NASH and liver fiber issues (p<0.01). This suggested that the genes in the gene set GS3 may play an important role in the progression of IBD and NAFLD.

### Identification and functional analysis of the common miRNAs of IBD and NAFLD

**Figure 12:**
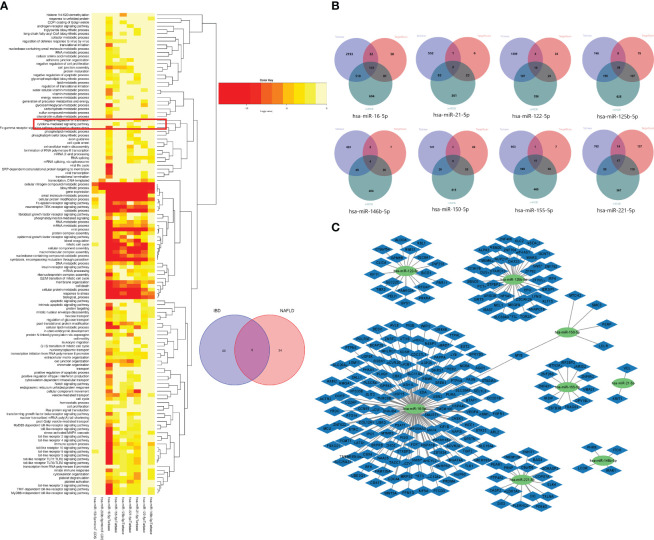
**(A)** GO functional enrichment analysis of nine common miRNAs. The box shows the chemokine-mediated signaling pathway. **(B)** Venn diagrams of the miRNA target genes predicted from the miRTarbase, miRDB, and TargetScan databases. **(C)** miRNA–mRNA network of MiRNAs and microRNAs.

Based on the Human MicroRNA Disease Database (HMDD)(https://www.cuilab.cn/hmdd) (23), it was found that 76 miRNAs were associated with IBD, and 43 miRNAs were associated with NAFLD (**Supplementary Table 1**). There were also nine overlapping miRNAs (hsa-miR-122-5p, hsa-miR-125b-5p, hsa-miR-146b-5p, hsa-miR-150-5p, hsa-miR155-5p, hsa-miR-16-5p, hsa-miR-200b-5p, hsa-miR-21-5p, and hsa-miR-2215p) between NAFLD and IBD. Using DIANA Tools mirPath v. 3 (https://dianalab.e-ce.uth.gr/html/mirpathv3/index.php?r=mirpath) performed KEGG enrichment analysis on these nine overlapping miRNAs and it was found that the “chemokine-mediated signaling pathway” and “interactions of viral proteins with cytokines and cytokine receptors” were involved in the biological processes in IBD and NAFLD (**Figure 12A**). This suggested that the miRNAs associated with the pathological mechanisms of IBD and NAFLD can also regulate the chemokine-mediated signaling pathway and the interaction of viral egg white with cytokines and cytokine receptors, thus confirming our findings. The nine potential target genes of the miRNAs were predicted based on the miRTarbase (https://mirtarbase.cuhk.edu.cn/~miRTarBase/miRTarBase_2022/php/index.php) (24), miRDB (https://mirdb.org/) (25), and TargetScan (https://www.targetscan.org/vert_80/) (26) databases. Unfortunately, the use of hsa-miR-200b-5p could not reveal the intersection of the target genes in the database (**Figure 12B**), while only hsa-miR-16-5p and hsa-miR-150-5p included overlapping genes. Finally, an miRNA–mRNA network was constructed (**Figure 12C**).

### Causal relationship between IL-17 and IBD and NAFLD

**Figure 13:**
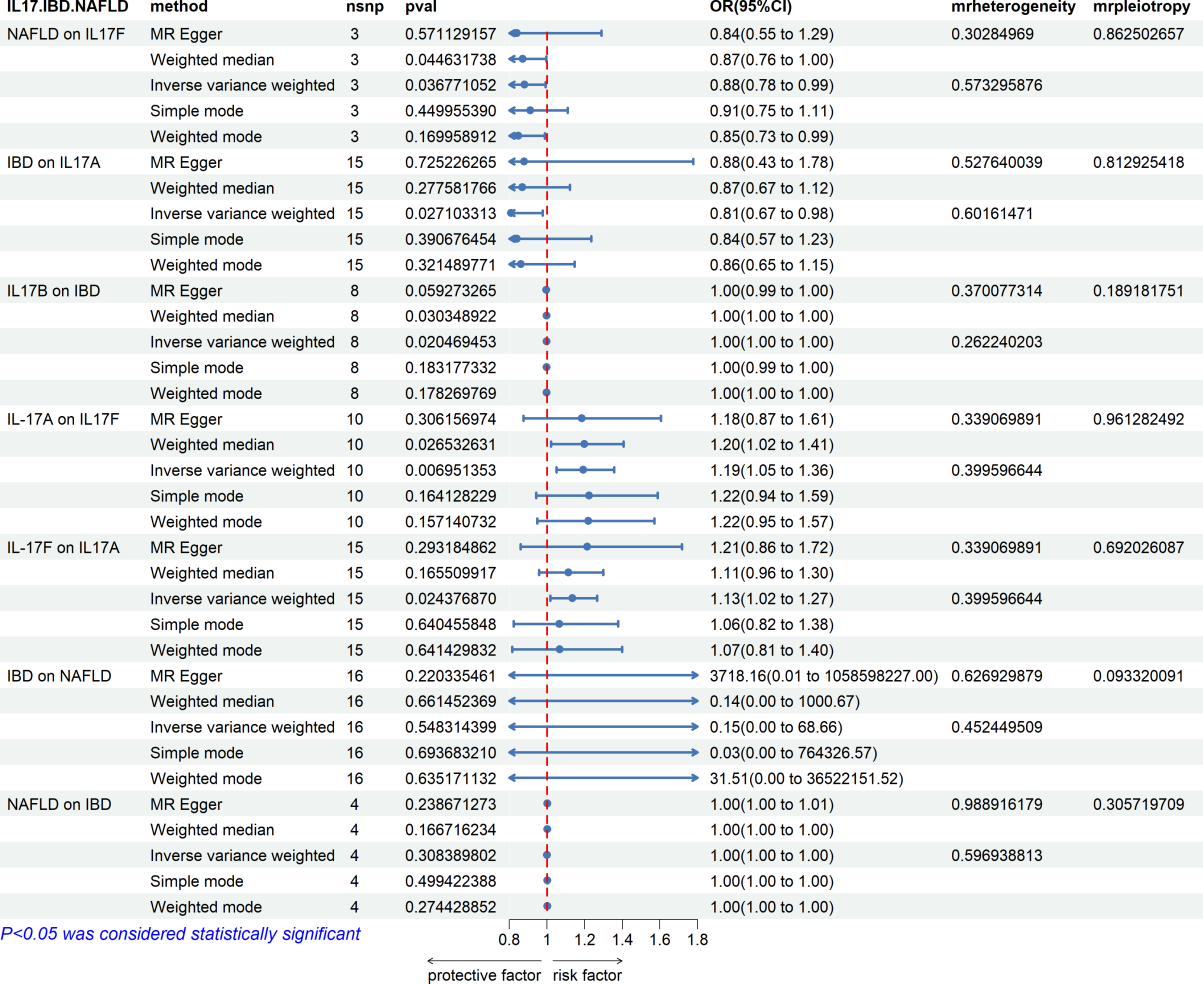
Associations with IBD and NAFLD. NAFLD is associated with IL-17F. IBD is associated with IL-17A and IL-17B, and IL-17A and IL-17F are bidirectionally correlated. There is no causal relationship between IBD and NAFLD.

From the study findings, we concluded that there is no causal relationship between IBD and NAFLD, but IL-17 has a causal relationship with IBD and NAFLD. IL-17A and IL-17B are related to IBD, while IL-17F is related to NAFLD also (**Figure 13**). Specifically, IBD was found to be negatively correlated with the expression level of IL-17A, while IL-17B was negatively correlated with IBD, and NAFLD was negatively correlated with the expression level of IL-17F. Finally, the expression levels of IL-17A and IL-17F were positively correlated in both directions.

## Discussion

As mentioned above, the incidence of NAFLD is high in IBD patients. The current view for why this is the case is that the decrease in beneficial bacteria and the increase in harmful bacteria that occur in the intestinal tract in IBD together with the intestinal inflammation mediate the impairment of the intestinal barrier function, leading to an increase in damaging factors entering into the circulation, and then leading to the occurrence of NAFLD through the “gut–liver axis” pathway. However, whether the gut microbiota or inflammatory factors, their exact role in IBD with NAFLD needs to be further studied. So far, no study has explored the susceptibility of NAFLD occurring in IBD at the level of gene expression and gene polymorphism. Through an in-depth analysis of the GEO database, this study found that genes related to the “chemokine-mediated signaling pathway” and “IL-17 signaling pathway” were present in modules highly related to IBD and NAFLD, and consistent results were obtained after repeated verification. In addition, this study also designed an miRNA–mRNA network. Although the common target genes of miRNAs associated with IBD and NAFLD did not have common intersection genes with GS1 and GS2, the enrichment results were still similar to those of the intersection genes. This implies that the biological pathway “chemokine-mediated signaling pathway” and the functional pathway “IL-17 signaling pathway” may play important roles in IBD and NAFLD. MR studies were performed and also supported the above results. This study specifically explored the causal relationship between IL-17 and the risk of IBD with NAFLD based on two-sample MR analysis of a large GWAS. This MR study showed that although there was no causal relationship between IBD and NAFLD, different subtypes of IL-17 had different causal relationships with IBD and NAFLD. MR is conceptually similar to a prospective randomized controlled trial (RCT) but enables reducing systematic biases, such as confounding and reverse causality, that commonly affect the results of traditional observational studies. The high accuracy of genotyping can effectively avoid the regression bias caused by detection errors. To ensure that SNPs were not associated with any confounding factors between IL-17, IBD, and NAFLD, we only selected participants from a European population. Finally, to ensure the stability of the results, we also performed MR-Egger regression tests, and no evidence of direction-level pleiotropy was observed.

In this study, the “IL-17-related pathway” and “chemokine-mediated signaling pathway” were at the core of the IBD/NAFLD comorbidity model. IL-17 is a cytokine secreted by T Helper 17 (TH17) cells, and chemokines are an important class of factors in IL-17-related pathways. IL-17 has opposite functions in the intestine. Under physiological conditions, IL-17 can promote the proliferation of intestinal epithelial cells (IECs), increase the expression of the multimeric Ig receptor (PIgR), promote the secretion of intestinal IgA, and secrete a variety of antimicrobial peptides to accelerate the healing of intestinal mucosal injuries, thereby enhancing intestinal barrier function (27). However, the intestinal flora of IBD patients is disordered, the abundance of Firmicutes is decreased, and harmful Gram-negative bacteria, such as *Proteobacteria, Bacteroidetes*, and *Actinobacteria*, are increased (28), These changes can induce the differentiation and proliferation of TH17 cells to a high level of pro-inflammatory type, and make IL-17 more pro-inflammatory (23, 24), First, IL-17 could synergistically activate NF-κB, ERK1/2, and p38 signaling pathways and cooperate with tumor necrosis factor-α to induce IL-17C secretion in the intestine and stimulate CCL20 expression in TH17 cells. Together with chemokine CCR6, TH17 cells can migrate to specific intestinal tissue sites to secrete IL-17 and aggravate IBD (25). Second, IL-17 alone or in combination with TNF-α acts on intestinal epithelial cells (IECs) to promote the secretion of inflammatory mediators, chemokines, and proteases, which will induce inflammatory responses and promote the recruitment, activation, and movement of neutrophils to target tissues, thereby causing intestinal mucosal injury (26). In addition to being affected by the gut microbiota, IL-17 secretion is also regulated by serum amyloid A (SAA) and can impair the integrity of the intestinal mucus barrier through this pathway (29). Among the possible mechanisms of IBD combined with NAFLD, it is speculated that intestinal inflammation and the barrier dysfunction in IBD may lead to an increased exposure of the liver to intestinal microorganisms and metabolites, leading to the occurrence of NAFLD and other liver diseases (30), which is known as the leaky gut theory. In this way, IL-17 and related chemokines can damage the intestinal barrier function and aggravate the severity of IBD (31). These results suggest that IL-17 and chemokine-related pathways are involved in the process of IBD inducing NAFLD (**Supplementary Figure 1**).

Some studies suggest that IBD-induced NAFLD can further aggravate the severity of IBD and increase the risk of hepatocellular carcinoma (HCC) (32–34). An epidemiology study found that people with metabolic dysfunction associated fatty liver (MAFLD) have a 12% increased risk of IBD (35). Metabolic syndrome, characterized by metabolic disorders, plays a major role in this (36), with IL-17 a key factor in the clinical manifestations of metabolic syndrome (37). Specifically, IL-17 and chemokines such as CXCR3 enhance the influence of the liver on peripheral insulin resistance by mediating liver inflammation, and promote the occurrence of NAFLD by participating in the process of metabolic syndrome (38–40). This is highly associated with the progression of NAFLD to NASH and liver fibrosis (41). In the process of NAFLD, liver adipose tissue can secrete a variety of adipokines (42), and these adipokines regulate the secretion of IL-17 by regulating the differentiation and function of Th17 cells. For example, leptin and resistin can directly induce the differentiation of TH17 cells into pro-inflammatory types and stimulate the secretion of IL-17 (43, 44). At the same time, adipokines can also affect the severity of NAFLD together with IL-17 by regulating the secretion of chemokines (45). A high fat diet (HFD) is one of the main causes of NAFLD, and the increased secretion of intestinal IL-17 and inflammatory chemokines under HFD conditions can lead to intestinal inflammation and damage and further aggravate adipose tissue inflammation (46–48). This also directly proves that metabolic disorders caused by IBD can cause NAFLD, affect the secretion regulation of IL-17 and chemokines, and may further cause intestinal inflammation and damage through IL-17-related pathways (**Supplementary Figure 2**).

Although this study and other MR results suggest that there is no causal relationship between IBD and NAFLD (49), there does seem to be a causal relationship between different subtypes of IL-17 and IBD and different subtypes of IBD (50). On this basis, this study further clarified that there is a causal relationship between different subtypes of IL-17 and NAFLD. According to the results of this study, chemokines are also important mediators of IL-17. Among them, the most representative chemokine in IBD combined with NAFLD is CCL2, and the secretion of CCL2 is regulated by IL-17 (51). CCL2 has been reported to be closely related to the severity of IBD and NAFLD (52, 53). At the same time, the expression of CCL2 in peripheral blood can also be used as an index to evaluate the severity of IBD and NAFLD (54). In an animal model, the levels of IL-17 and CCL2 in the liver and intestinal tissues of mice treated with HF+DSS were higher than those of mice treated with HF or DSS alone, and were also found to be related to liver fibrosis (55). This suggests that liver and intestinal lesions can interact, and that IL-17 and CCL2 may be the key mediators. The above evidence suggests that even if there is no causal relationship between IBD and NAFLD, IL-17 and chemokines cannot be ignored in this particular disease manifestation.

To further determine the role of chemokines in the pathogenesis of IBD combined with NAFLD, a miRNA–mRNA network was constructed using the HMDD, miRTarbase, miRDB, and TargetScan databases. It was found that the target genes of the common miRNAs did not share intersection genes with GS1 and GS2, but were still enriched in “chemokine-mediated signaling pathways”, which may be related to the indirect interactions between genes. This also indicates that IL-17 may affect the special disease manifestation of IBD combined with NAFLD through a pathway related to chemokines.

The results of the MR analysis in this study suggest that IL-17A and IL-17B may be protective factors for IBD. In the case of IL-17A, this accords with some prior animal experiments and clinical observation experiments that also suggested that IL-17A may have a protective effect on IBD (56, 57). This study can support this view to a certain extent, but there are also studies that have reported that IL-17A is not related to IBD. It has been suggested that it may even promote the occurrence of IBD (58), which needs to be verified by further experiments. However, there are few studies on IL-17B in IBD. One of the few existing studies suggested that IL-17B regulates the response of colonic myeloid cells to inhibit colitis (59), which is the same as the results of this study. IL-17A is generally considered to play a promoting role in NAFLD, while IL-17F has been reported to play a role in preventing liver function damage in NAFLD (60), which is also consistent with the results of this study. Although the relationship between IL-17A and IL-17F has not been discussed so far, the results of this study suggest a causal relationship between IL-17A and IL-17F at the genetic level, and IL-17A and IL-17F were confirmed to play roles in IBD and NAFLD, respectively. Therefore, we hypothesize that the bidirectional promotion relationship between IL-17A and IL-17F may play an important role in the special disease manifestation of IBD combined with NAFLD.

Among the miRNAs shared by IBD and NAFLD identified in this study, hsa-miR-122-5p has been reported to be a serum marker for the diagnosis of NAFLD (61), while has-miR-146b-5p is associated with different subtypes of UC (62). At the same time, hsa-miR-150-5p and hsa-Mir-150-5p were used as the related miRNAs of liver fibrosis (63). We speculate that has-miR-146b-5p may play a role in the pathogenesis of IBD combined with NAFLD, but this needs to be confirmed by further studies.

In conclusion, this study established a comorbidity model to explain the underlying mechanism of IBD combined with NAFLD, identified the cytokine IL-17-mediated chemokine-related pathway as the core pathway in the IBD combined with NAFLD comorbidity model, and found that miRNAs may also play a role in IBD with NAFLD. The use of MR to clarify the causal relationship between IL-17 and IBD and NAFLD helped identify it as a potential target for the treatment of IBD combined with NAFLD.

## Data availability statement

The original contributions presented in the study are included in the article/**Supplementary Material**. Further inquiries can be directed to the corresponding author.

## Author contributions

ZW: Conceptualization, Data curation, Formal analysis, Investigation, Methodology, Software, Writing – original draft, Writing – review & editing. JW: Conceptualization, Project administration, Supervision, Writing – original draft, Writing – review & editing.
