# Supplementary material for: Exploration of the core pathway of inflammatory bowel disease complicated with metabolic fatty liver and two-sample Mendelian randomization study of the causal relationships behind the disease
Source: Front Immunol. 2024 Apr 18;15:1375654. doi: 10.3389/fimmu.2024.1375654 (PMC11063260; doi:10.3389/fimmu.2024.1375654)
Supplement: Supplementary file 1 [file DataSheet_1.docx]

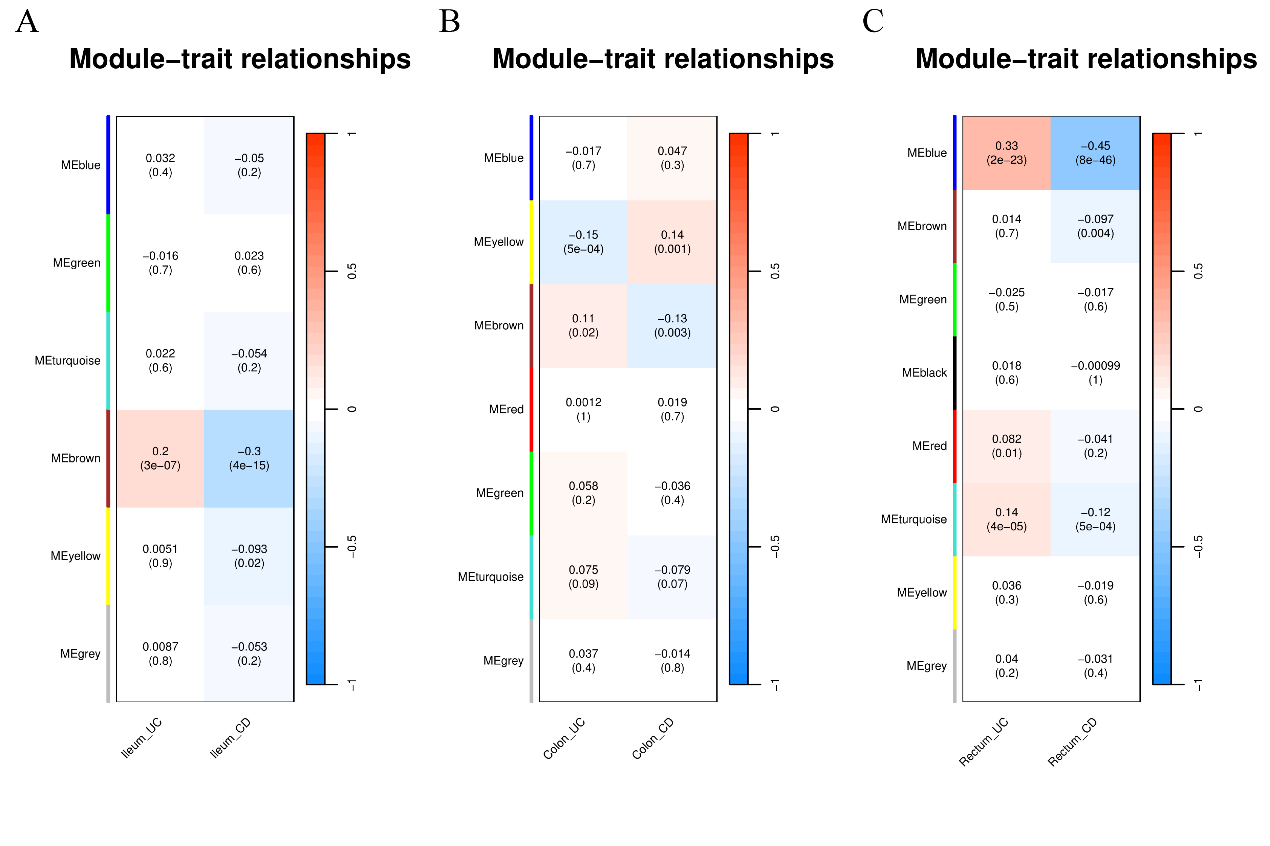


**Supplementary Figure 1.** A, Results of WGCNA performed separately for UC and CD in ileal tissue; B, Results of WGCNA performed separately for UC and CD in colon tissue; C, Results of WGCNA performed separately for UC and CD in rectal tissue.


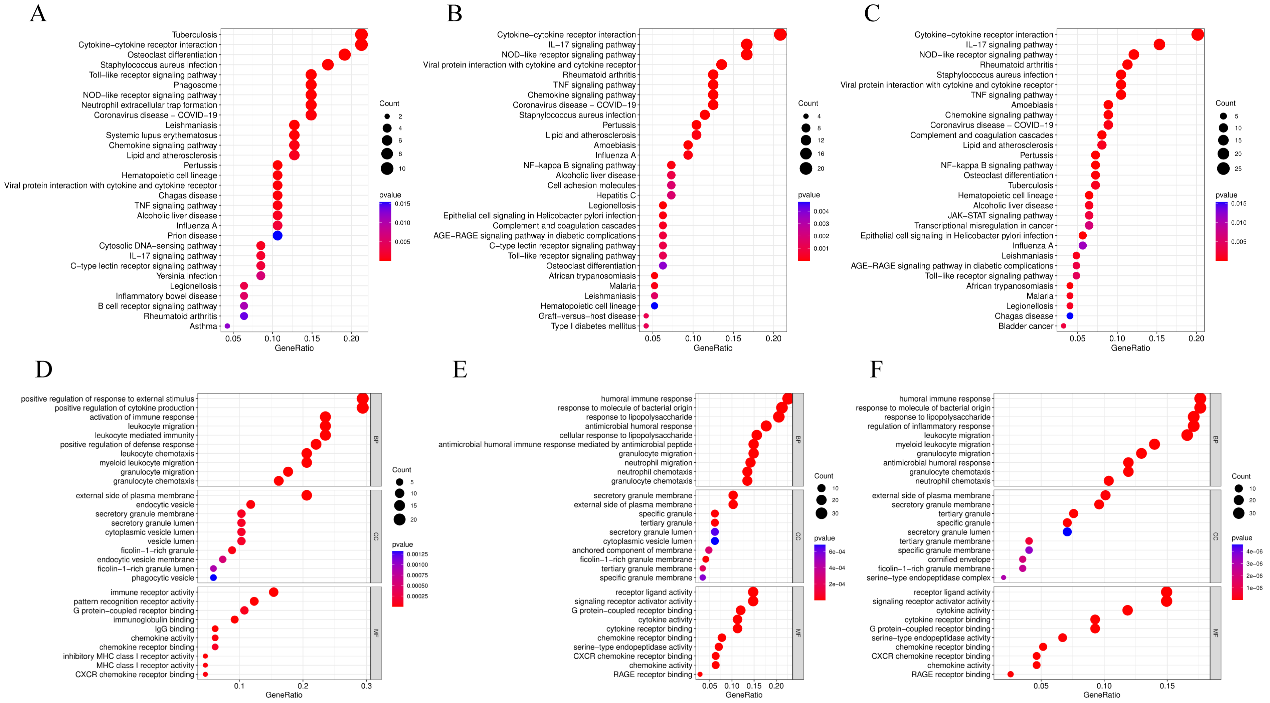


**Supplementary Figure 2.** AD, KEGG and GO enrichment analysis of green modules in ileal tissues; BE, KEGG and GO enrichment analysis of the brown module of colon tissue; CF, KEGG and GO enrichment analysis of the red module of rectal tissue.


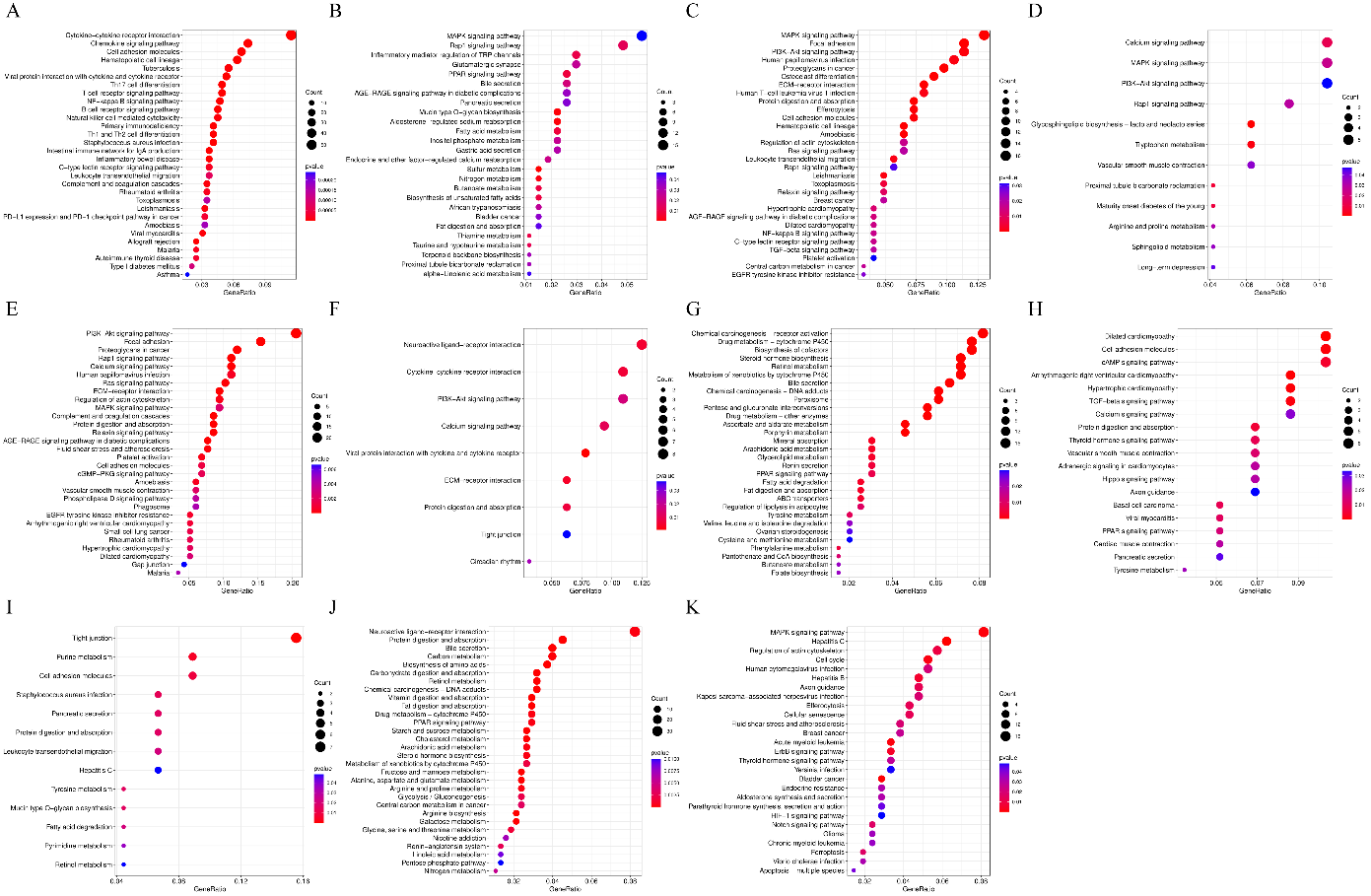


**Supplementary Figure 3.** GSE193677 performed KEGG enrichment analysis of modules identified by WGCNA analysis


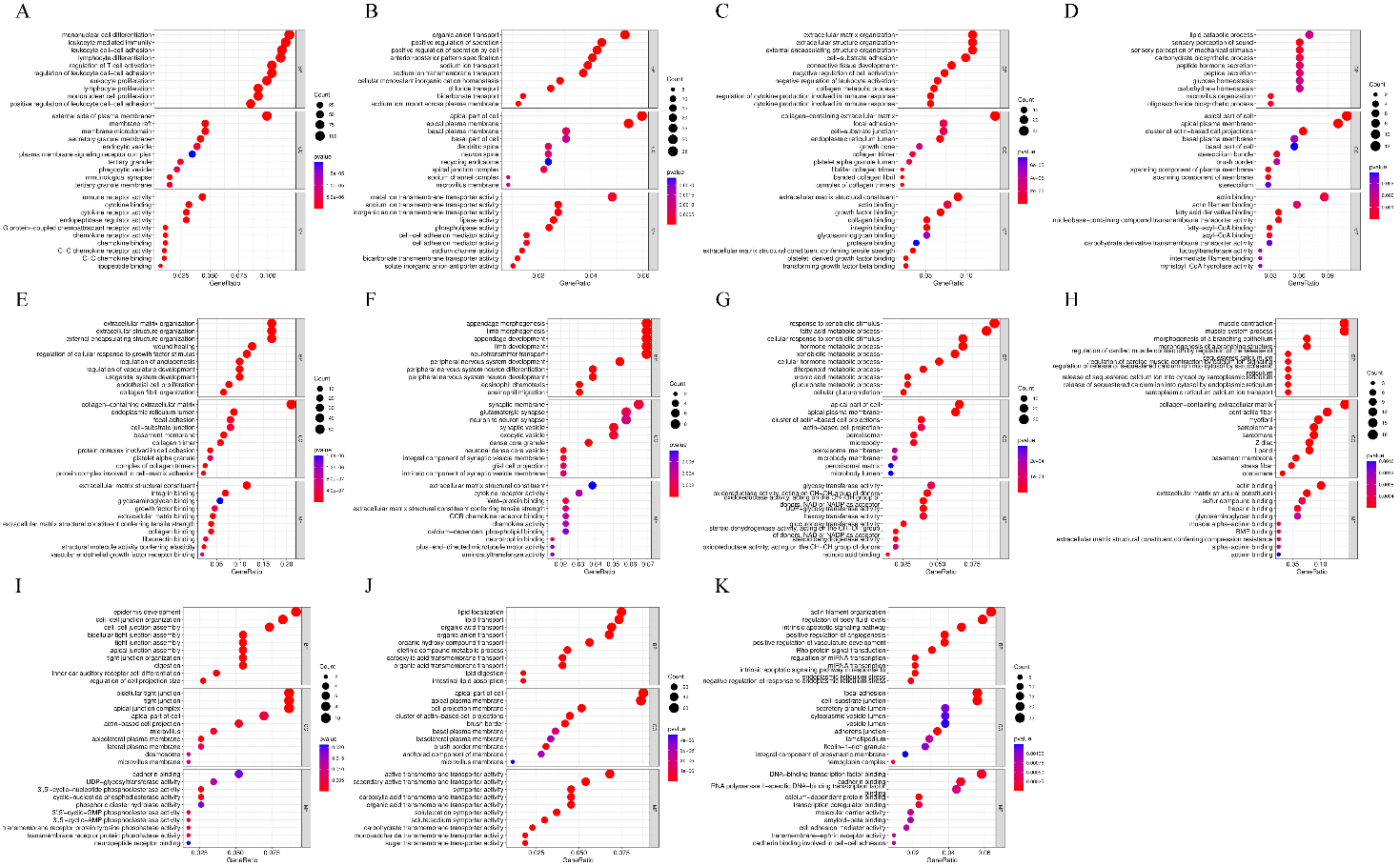


**Supplementary Figure 4.** GSE193677 performed GO enrichment analysis of modules identified by WGCNA analysis


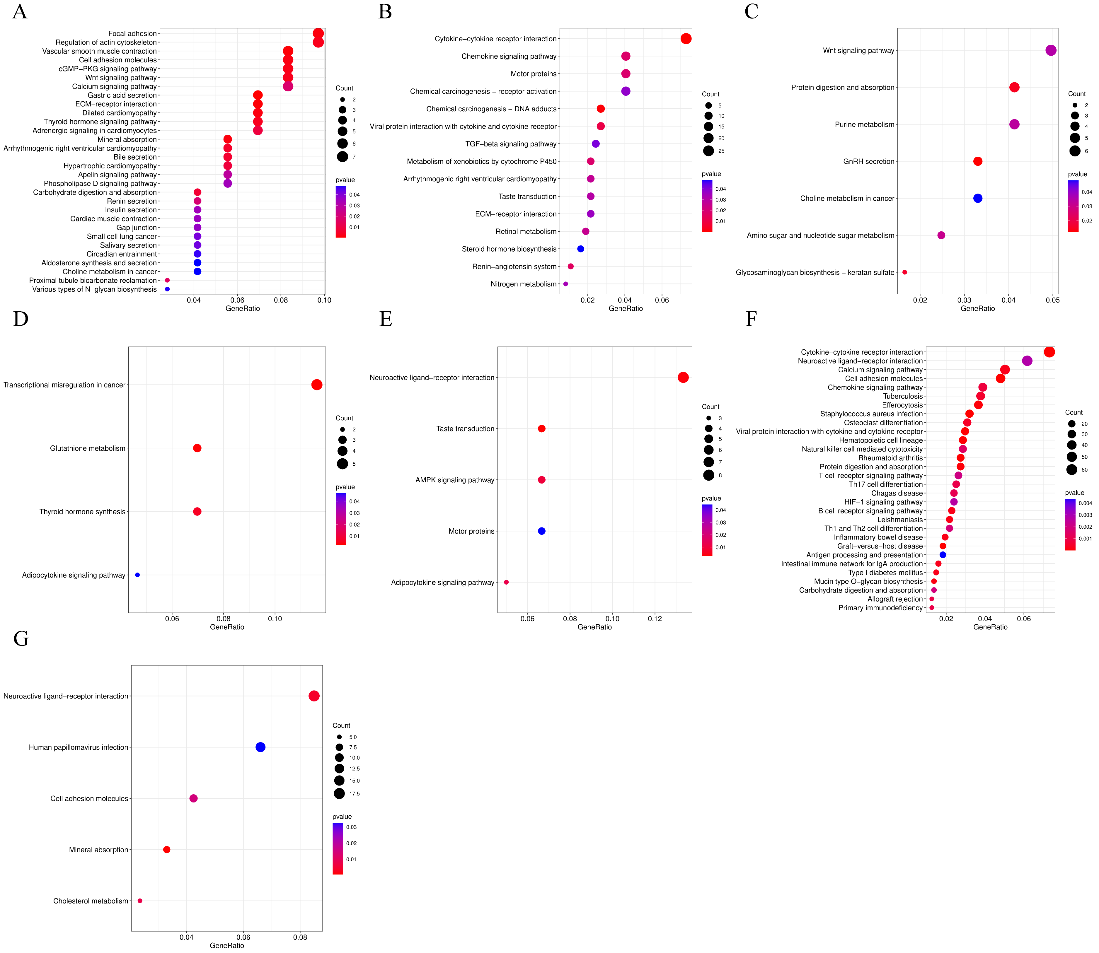


**Supplementary Figure 5.** GSE126848 performed KEGG enrichment analysis of modules identified by WGCNA analysis


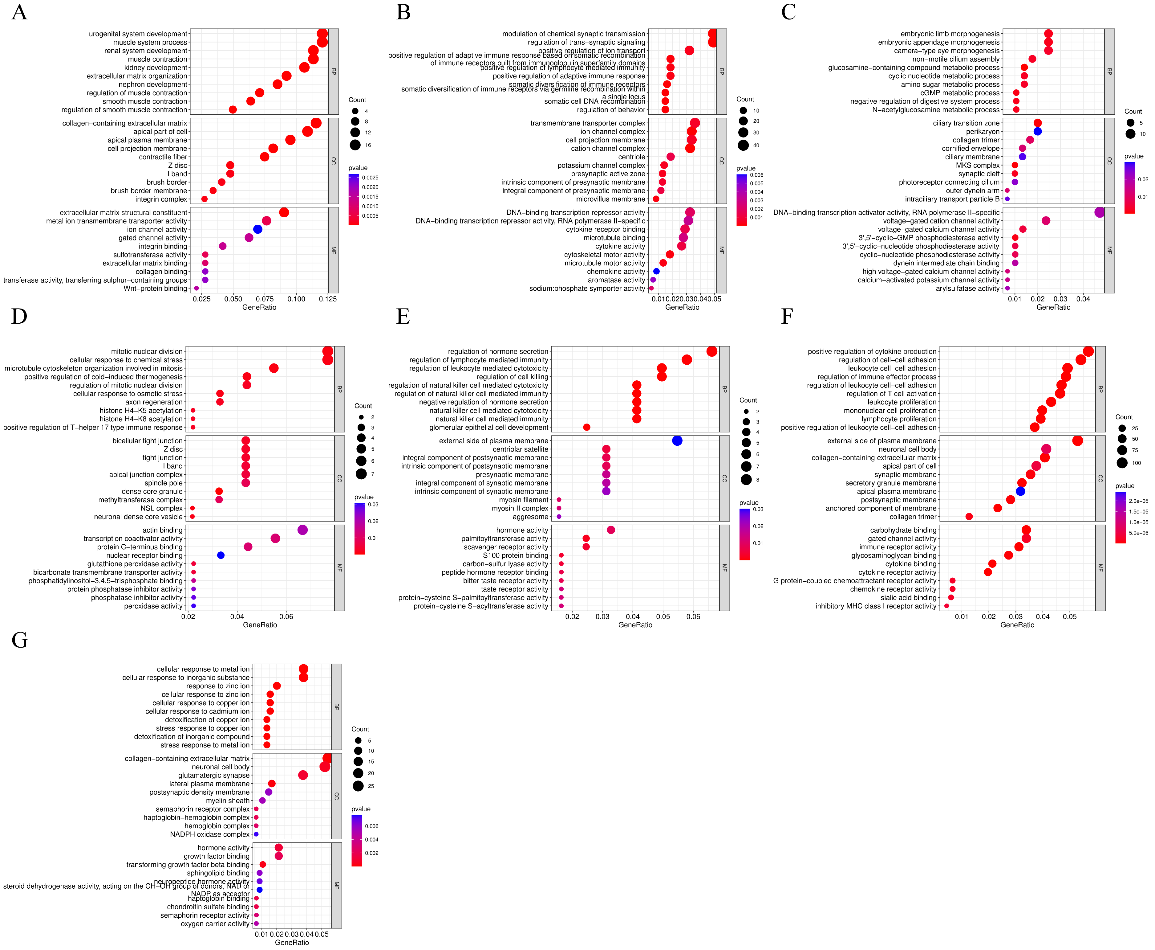


**Supplementary Figure 6.** GSE126848 performed GO enrichment analysis of modules identified by WGCNA analysis


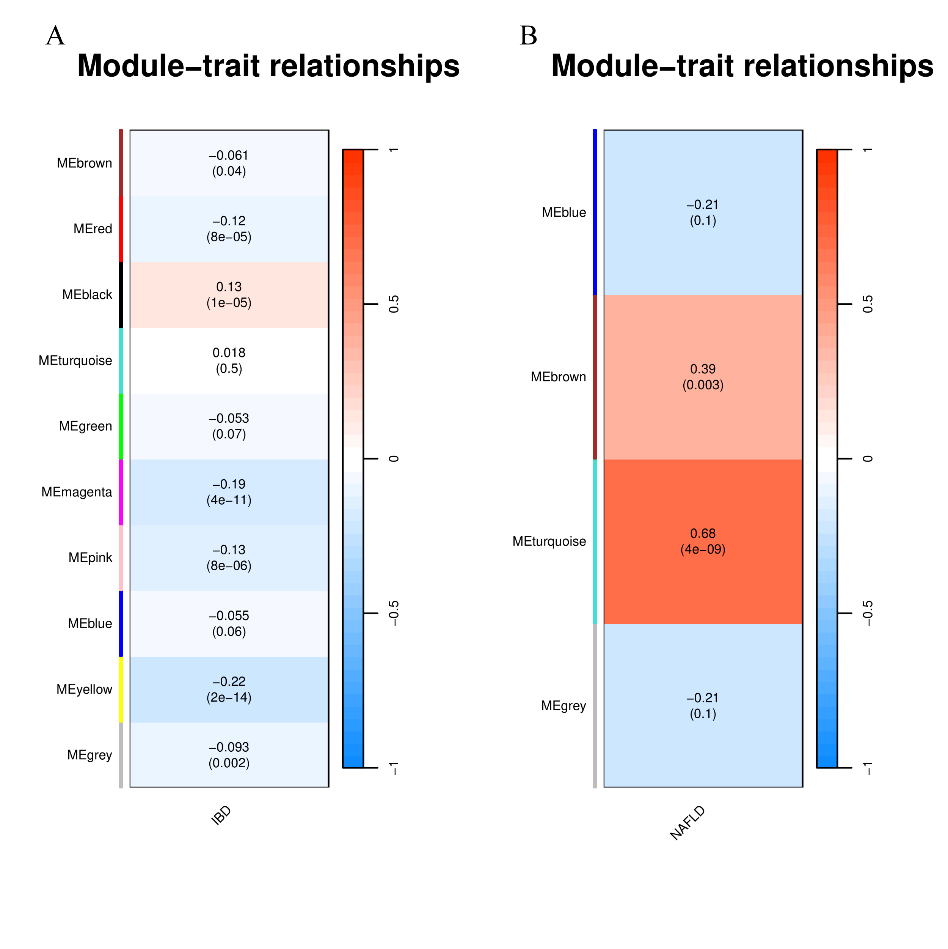


**Supplementary Figure 7.** A, WGCNA results for samples from IBD patients only; B, WGCNA results for samples from patients with NAFLD only


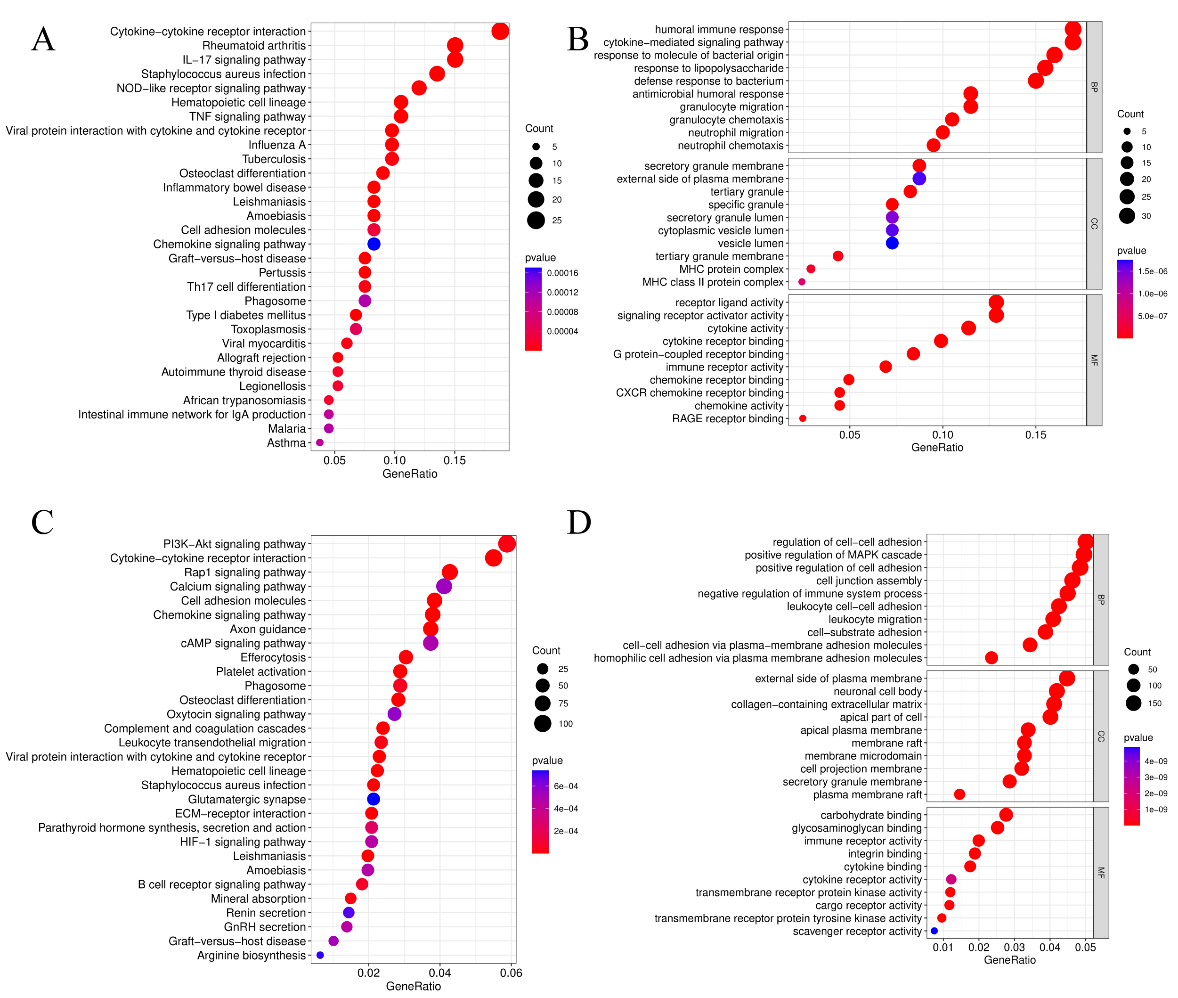


**Supplementary Figure 8.** AB, KEGG and GO enrichment analysis results of the black module in WGCNA results of IBD patients; CD, KEGG and GO enrichment analysis results of turquoise module in WGCNA results of NAFLD patients
