# Supplementary material for: Exploration of the core pathway of inflammatory bowel disease complicated with metabolic fatty liver and two-sample Mendelian randomization study of the causal relationships behind the disease
Source: Front Immunol. 2024 Apr 18;15:1375654. doi: 10.3389/fimmu.2024.1375654 (PMC11063260; doi:10.3389/fimmu.2024.1375654)
Supplement: Supplementary file 3 [file DataSheet_3.docx]

**STROBE-MR checklist of recommended items to address in reports of Mendelian randomization studies**^1^ ^2^

| **Item No.** | **Section** | **Checklist item** | **Page No.** | **Relevant text from manuscript** |
| --- | --- | --- | --- | --- |
| 1 | **TITLE and ABSTRACT** | Indicate Mendelian randomization (MR) as the study’s design in the title and/or the abstract if that is a main purpose of the study | 1 | Exploration of the core pathway of inflammatory bowel disease complicated with metabolic fatty liver and two-sample Mendelian randomization study of the causal relationships behind the disease |
|  | **INTRODUCTION** |  |  |  |
| 2 | **Background** | Explain the scientific background and rationale for the reported study. What is the exposure? Is a potential causal relationship between exposure and outcome plausible? Justify why MR is a helpful method to address the study question | 6 | MR has become popular in recent years as a reliable method for inferring potential causal relationships, and has been applied using single nucleotide polymorphisms (SNPs) as instrumental variables (IVs) to assess the causal relationships between exposure factors and outcomes. |
| 3 | **Objectives** | State specific objectives clearly, including pre-specified causal hypotheses (if any). State that MR is a method that, under specific assumptions, intends to estimate causal effects | 6 | Of interest in the present study, it was believed that MR using genetic variants strongly associated with different exposure factors as instrumental variables could infer the causal relationships between the exposure factors and study outcomes. |
|  | **METHODS** |  |  |  |
| 4 | **Study design and data sources** | Present key elements of the study design early in the article. Consider including a table listing sources of data for all phases of the study. For each data source contributing to the analysis, describe the following: |  |  |
|  | a) | Setting: Describe the study design and the underlying population, if possible. Describe the setting, locations, and relevant dates, including periods of recruitment, exposure, follow-up, and data collection, when available. |  |  |
|  | b) | Participants: Give the eligibility criteria, and the sources and methods of selection of participants. Report the sample size, and whether any power or sample size calculations were carried out prior to the main analysis | 9 | The IL-17, IBD, and NAFLD datasets selected for analysis, and the exposure and outcome data are detailed in Table 1. |
|  | c) | Describe measurement, quality control and selection of genetic variants | 9 | The IL-17, IBD, and NAFLD datasets selected for analysis, and the exposure and outcome data are detailed in Table 1. |
|  | d) | For each exposure, outcome, and other relevant variables, describe methods of assessment and diagnostic criteria for diseases | 9 | Two-sample and bidirectional MR were applied to explore the causal relationship between the identified target of interest from the above analysis, which was interleukin-17 (IL-17), and the risk of IBD and NAFLD. |
|  | e) | Provide details of ethics committee approval and participant informed consent, if relevant |  |  |
| 5 | **Assumptions** | Explicitly state the three core IV assumptions for the main analysis (relevance, independence and exclusion restriction) as well assumptions for any additional or sensitivity analysis | 9 | A genome-wide significance level of p < 5×10^-8^, minor allele frequency < 0.01, and clumping algorithm with a cutoff of r2 = 0.001 and kb = 10000 were used to avoid linkage disequilibrium (LD). |
| 6 | **Statistical methods: main analysis** | Describe statistical methods and statistics used |  |  |
|  | a) | Describe how quantitative variables were handled in the analyses (i.e., scale, units, model) | 9 |  |
|  | b) | Describe how genetic variants were handled in the analyses and, if applicable, how their weights were selected | 9 |  |
|  | c) | Describe the MR estimator (e.g. two-stage least squares, Wald ratio) and related statistics. Detail the included covariates and, in case of two-sample MR, whether the same covariate set was used for adjustment in the two samples | 9 | The strength of the genetic variance of each exposure was estimated by R2 and F-statistics. IVs with F-statistics over 10 were used for subsequent analyses to avoid weak instrument bias. |
|  | d) | Explain how missing data were addressed | 9 |  |
|  | e) | If applicable, indicate how multiple testing was addressed |  |  |
| 7 | **Assessment of assumptions** | Describe any methods or prior knowledge used to assess the assumptions or justify their validity | 10 | Inverse-variance weighted (IVW) meta-analysis with a multiplicative random model was used as the major analysis methodology for causal estimation. Cochran’s Q test was performed to detect heterogeneity among the genetic variants. |
| 8 | **Sensitivity analyses and additional analyses** | Describe any sensitivity analyses or additional analyses performed (e.g. comparison of effect estimates from different approaches, independent replication, bias analytic techniques, validation of instruments, simulations) | 10 | The weighted median model, MR−Egger regression model, and Mendelian randomization pleiotropy residual sum and outlier were performed for the sensitivity analyses and to detect the existence of horizontal pleiotropy that would violate the main MR assumptions. |
| 9 | **Software and pre-registration** |  |  |  |
|  | a) | Name statistical software and package(s), including version and settings used | 10 | The MR analysis was based on the "TwoSampleMR" software package and used inverse variance weighting (IVW) to assess the association between IL-17 and the risk of IBD and NAFLD. |
|  | b) | State whether the study protocol and details were pre-registered (as well as when and where) | 9 | All the data in this study were available and used in an open database. |
|  | **RESULTS** |  |  |  |
| 10 | **Descriptive data** |  |  |  |
|  | a) | Report the numbers of individuals at each stage of included studies and reasons for exclusion. Consider use of a flow diagram | 16 | From the study findings, we concluded that there is no causal relationship between IBD and NAFLD, but IL-17 has a causal relationship with IBD and NAFLD. IL-17A and IL-17B are related to IBD, while IL-17F is related to NAFLD also (Figure 13). |
|  | b) | Report summary statistics for phenotypic exposure(s), outcome(s), and other relevant variables (e.g. means, SDs, proportions) | 16 | Figure 13 |
|  | c) | If the data sources include meta-analyses of previous studies, provide the assessments of heterogeneity across these studies |  |  |
|  | d) | For two-sample MR:  i.  Provide justification of the similarity of the genetic variant-exposure associations between the exposure and outcome samples  ii.  Provide information on the number of individuals who overlap between the exposure and outcome studies | 16 | Specifically, IBD was found to be negatively correlated with the expression level of IL-17A, while IL-17B was negatively correlated with IBD, and NAFLD was negatively correlated with the expression level of IL-17F. Finally, the expression levels of IL-17A and IL-17F were positively correlated in both directions. |
| 11 | **Main results** |  |  |  |
|  | a) | Report the associations between genetic variant and exposure, and between genetic variant and outcome, preferably on an interpretable scale | 16 | Figure 13 |
|  | b) | Report MR estimates of the relationship between exposure and outcome, and the measures of uncertainty from the MR analysis, on an interpretable scale, such as odds ratio or relative risk per SD difference | 16 | From the study findings, we concluded that there is no causal relationship between IBD and NAFLD. |
|  | c) | If relevant, consider translating estimates of relative risk into absolute risk for a meaningful time period | 16 | IL-17 has a causal relationship with IBD and NAFLD. IL-17A and IL-17B are related to IBD, while IL-17F is related to NAFLD also. |
|  | d) | Consider plots to visualize results (e.g. forest plot, scatterplot of associations between genetic variants and outcome versus between genetic variants and exposure) | 16 | Figure 13 |
| 12 | **Assessment of assumptions** |  |  |  |
|  | a) | Report the assessment of the validity of the assumptions | 16 | Figure 13 |
|  | b) | Report any additional statistics (e.g., assessments of heterogeneity across genetic variants, such as *I^2^*, Q statistic or E-value) | 16 | Figure 13 |
| 13 | **Sensitivity analyses and additional analyses** |  |  |  |
|  | a) | Report any sensitivity analyses to assess the robustness of the main results to violations of the assumptions | 16 | From the study findings, we concluded that there is no causal relationship between IBD and NAFLD, but IL-17 has a causal relationship with IBD and NAFLD. IL-17A and IL-17B are related to IBD, while IL-17F is related to NAFLD also. |
|  | b) | Report results from other sensitivity analyses or additional analyses | 16 | Figure 13 |
|  | c) | Report any assessment of direction of causal relationship (e.g., bidirectional MR) | 16 | Figure 13 |
|  | d) | When relevant, report and compare with estimates from non-MR analyses | no |  |
|  | e) | Consider additional plots to visualize results (e.g., leave-one-out analyses) | no |  |
|  | **DISCUSSION** |  |  |  |
| 14 | **Key results** | Summarize key results with reference to study objectives | 17 | MR studies were performed and also supported the above results. |
| 15 | **Limitations** | Discuss limitations of the study, taking into account the validity of the IV assumptions, other sources of potential bias, and imprecision. Discuss both direction and magnitude of any potential bias and any efforts to address them | 21 | Although the relationship between IL-17A and IL-17F has not been discussed so far |
| 16 | **Interpretation** |  |  |  |
|  | a) | Meaning: Give a cautious overall interpretation of results in the context of their limitations and in comparison with other studies | 21-22 | Therefore, we hypothesize that the bidirectional promotion relationship between IL-17A and IL-17F may play an important role in the special disease manifestation of IBD combined with NAFLD. |
|  | b) | Mechanism: Discuss underlying biological mechanisms that could drive a potential causal relationship between the investigated exposure and the outcome, and whether the gene-environment equivalence assumption is reasonable. Use causal language carefully, clarifying that IV estimates may provide causal effects only under certain assumptions | 21 | One of the few existing studies suggested that IL-17B regulates the response of colonic myeloid cells to inhibit colitis (59), which is the same as the results of this study. |
|  | c) | Clinical relevance: Discuss whether the results have clinical or public policy relevance, and to what extent they inform effect sizes of possible interventions | 21 | IL-17A is generally considered to play a promoting role in NAFLD, while IL-17F has been reported to play a role in preventing liver function damage in NAFLD. |
| 17 | **Generalizability** | Discuss the generalizability of the study results (a) to other populations, (b) across other exposure periods/timings, and (c) across other levels of exposure | 21 | The results of the MR analysis in this study suggest that IL-17A and IL-17B may be protective factors for IBD. |
|  | **OTHER INFORMATION** |  |  |  |
| 18 | **Funding** | Describe sources of funding and the role of funders in the present study and, if applicable, sources of funding for the databases and original study or studies on which the present study is based |  |  |
| 19 | **Data and data sharing** | Provide the data used to perform all analyses or report where and how the data can be accessed, and reference these sources in the article. Provide the statistical code needed to reproduce the results in the article, or report whether the code is publicly accessible and if so, where | 23 | All data generated or analyzed during this study are included in this published article. |
| 20 | **Conflicts of Interest** | All authors should declare all potential conflicts of interest | 23 | Not applicable |

This checklist is copyrighted by the Equator Network under the Creative Commons Attribution 3.0 Unported (CC BY 3.0) license.

1. Skrivankova VW, Richmond RC, Woolf BAR, Yarmolinsky J, Davies NM, Swanson SA, et al. Strengthening the Reporting of Observational Studies in Epidemiology using Mendelian Randomization (STROBE-MR) Statement. JAMA. 2021;under review.

2. Skrivankova VW, Richmond RC, Woolf BAR, Davies NM, Swanson SA, VanderWeele TJ, et al. Strengthening the Reporting of Observational Studies in Epidemiology using Mendelian Randomisation (STROBE-MR): Explanation and Elaboration. BMJ. 2021;375:n2233.
